# Phytogenic supplementation mitigates necrotic enteritis in broilers through modulation of gut microbiota, short-chain fatty acids, and nutrient-related gene expression

**DOI:** 10.1016/j.psj.2026.107955

**Published:** 2026-10-11

**Authors:** Most Khairunnesa, Alip Kumar, Shu-Biao Wu, Thi Thu Hao Van, Kowsig Palanisamy, Kosar Gharib-Naseri

**Affiliations:** a School of Environmental and Rural Science, University of New England, Armidale, NSW, 2351, Australia; b School of Science, RMIT University, Bundoora, VIC, 3083, Australia; c EW Nutrition, Hogenbögen 1, Visbek, 49429, Germany

**Keywords:** Phytogenic blend, Microbiota, Broiler, Necrotic enteritis, *Clostridium perfringens*

## Abstract

With the withdrawal of antibiotic growth promoters, phytogenic compounds have gained attention as potential alternatives for enhancing gut health and modulating the intestinal microbiota. This study investigated the effects of a phytogenic blend (**PHB**) containing thymol, oleic acid, capric acid, and refined sunflower fat on cecal short-chain fatty acids (**SCFA**), jejunal gene expression, and cecal microbiota composition in broiler chickens under necrotic enteritis (**NE**) challenge. A total of 450 d-old mixed-sex Cobb 500 broiler chicks were randomly allocated to three treatments: non-challenged control without additives (**NC**), NE-challenged control without additives (**CC**), and NE-challenged supplemented with 0.01% phytogenic blend (**PHB**). Birds in challenge groups were orally inoculated with *Eimeria* spp. on d9 and *Clostridium perfringens* on d14 to induce NE. Data were analyzed using one-way ANOVA, and mean separation was performed by Tukey’s test (*P* < 0.05). Microbiota composition was assessed through 16S rRNA sequencing, and downstream microbiome analyses and visualization were performed using R version 4.6.1 (R Core Team, 2026) in RStudio. The NE challenge upregulated interferon-gamma (***IFN-γ***; *P* < 0.05), downregulated claudin 5 (***CLDN5***; *P* < 0.05) and immunoglobulin M (***IgM***; *P* < 0.05) genes, and significantly increased iso-butyric, iso-valeric, and valeric acid concentrations compared to the NC group (*P* < 0.05). Moreover, it reduced microbial diversity (*P* < 0.05) and increased the abundance of potentially pathogenic genera, including *Clostridium sensu stricto* 1, and *Escherichia Shigella*. The dietary PHB significantly reduced the concentrations of iso-butyric, iso-valeric, and valeric acids, and increased lactic and succinic acids (*P* < 0.05), and upregulated *APN* (*P* < 0.05). l-type amino acid transporter 1 (***LAT1***) showed an intermediate response in the PHB group and did not reach statistical significance compared to CC (*P* > 0.05). Furthermore, PHB did not significantly affect *IFN-γ, CLDN5*, or *IgM* levels (*P* > 0.05). Microbiota analysis revealed distinct β-diversity clustering between CC and PHB groups and increased abundance of beneficial bacterial taxa in the PHB group, including *Tyzzerella, Lactobacillus, Anaerostipes, Ruminococcus_torques_*group, *Butyricicoccus pullicaecorum*, with an elevated Firmicutes-to-Bacteroidetes ratio. These findings suggest that PHB supplementation alleviated NE-induced gut dysbiosis and metabolic disruption by promoting beneficial bacteria, improving SCFA profiles, and enhancing mucosal gene expression related to digestion.

## Introduction

Gut health is a critical area of research in both humans and animals, as it is influenced by diet, microbiota composition, and pathogen exposure, all of which can impair nutrient utilization and overall health (Choct, 2009). In poultry, compromised gut function often predisposes birds to enteric infections, among which necrotic enteritis (**NE**) remains one of the most economically significant diseases. NE is characterized by necrotic lesions in the small intestinal mucosa and leads to considerable performance losses and mortality (Kaldhusdal and Hofshagen, 1992). The primary causative agent of NE is *Clostridium perfringens*, a gram-positive, anaerobic, spore-forming bacterium (Keyburn et al., 2010). Among the six known toxin types of *C. perfringens*, type G, capable of producing necrotic enteritis toxin B (NetB), is the predominant strain responsible for NE outbreaks in poultry (Rood et al., 2018).

NE can occur in two forms: the clinical form is characterized by diarrhea, depression, and high mortality rates reaching up to 50% (Gholamiandehkordi et al., 2007), and the subclinical form (**SNE**) progresses without visible symptoms but causes chronic intestinal damage, reducing nutrient digestion and absorption and leading to significant economic losses through poor performance (Khalique et al., 2020). For many years, the use of in-feed antibiotics effectively controlled NE. However, concerns about antimicrobial resistance and drug residues in animal products have led to global restrictions, including the European Union's ban from 2006 on antibiotics as feed additives (Castanon, 2007). This has contributed to an increased occurrence of NE, particularly in "antibiotic-free" production systems, which are now widely adopted due to consumers' demand (Bueno et al., 2023). To address this challenge, research has focused on identifying safe and effective non-antibiotic alternatives to maintain gut health. Among these, phytogenic feed additives (**PFA**), plant-derived products such as herbal extracts and essential oils, have gained significant attention for their multifunctional benefits (Redondo et al., 2014). Several studies have demonstrated that phytogenic compounds can favorably modulate gut microbiota, increase the expression of tight junction and immune-related genes, and enhance gut barrier function (Alharthi et al., 2023; Ibrahim et al., 2020; Paraskeuas and Mountzouris, 2019). The phytogenic blend (**PHB**) is a commercial PFA, composed of thymol, oleic acid, capric acid, and refined sunflower fat. Although direct scientific evidence on PHB is limited, its key components have been individually associated with improved gut function. Thymol demonstrates strong anti-NE efficacy either in combination with carvacrol, reducing intestinal lesion severity and mortality while increasing the beneficial *Lactobacillus* population, or with fatty acids, enhancing epithelial protection against NE damage (Timbermont et al., 2010; Yin et al., 2017). Moreover, thymol enhanced the intestinal barrier response and *Lactobacillus* population under *Salmonella typhimurium* infection in broilers (Ibrahim et al., 2021). Beyond broilers, thymol in combination with cinnamaldehyde also upregulated intestinal barrier genes in laying hens (Wang et al., 2022), enhanced immune responses, and promoted beneficial bacteria in turkeys when combined with formic acid (Champati et al., 2024).

Taken together, these findings suggest that phytogenic compounds may improve intestinal health through complementary actions on the microbiota, epithelial barrier, and immune system. However, limited information is available on how combined phytogenic compounds perform under NE challenge in broilers. Based on existing evidence, it is hypothesized that dietary supplementation with PHB would mitigate NE-induced intestinal damage in broilers by modulating the gut microbiota, improving short-chain fatty acids (**SCFA**) profiles, and regulating genes associated with gut integrity and immune function. Therefore, the objective of the current study was to evaluate the efficacy of PHB supplementation in alleviating the adverse effects of NE on cecal microbiota, SCFA, and gene expression in broiler chickens.

## Materials and methods

### Animal ethics

The experimental protocols used in this study were approved by the Animal Ethics Committee at the University of New England, Armidale, NSW 2351, Australia (Approval No. ARA23-094). All procedures complied with the Australian Code for the Care and Use of Animals for Scientific Purposes and were accredited by the Australian Bureau of Animal Health (NHMRC, 2013).

### Feed additives

Ventar D (EW Nutrition GmbH, Germany) is a commercial PHB that contains encapsulated active compounds, including thymol, oleic acid, capric acid, and refined sunflower fat, which possess antimicrobial, anti-inflammatory, and antioxidant properties.

### Experimental design and diets

A total of 450 d-old mixed-sex Cobb 500 broiler chicks were used in this study. The d-old chicks were obtained from Baiada Hatchery, Tamworth, NSW, and allocated to 30 floor pens, with pen weights balanced across treatments. The experiment followed a completely randomized design with three treatments; each replicated 10 times, with 15 birds per replication, resulting in 450 birds in total. The treatments included: (1) non-challenged control without additives (**NC**); (2) NE-challenged control without additives (**CC**); and (3) NE-challenged group supplemented with phytogenic blend at 0.01% (**PHB**) (Table 1). Birds were managed in an environmentally controlled facility, bedded with softwood shavings (approximately 8 cm depth). Each pen was equipped with one tube feeder and three nipple drinkers to provide ad libitum access to feed and water. Cobb 500 management guidelines were followed for temperature, humidity, and lighting schedule. The temperature was set at 33°C for the first three days and then gradually reduced by 2°C every 2 days until it reached 22°C by d21 (Cobb 500, 2022b). All diets were formulated using corn-sorghum-wheat-soybean meal as the primary ingredients, supplemented with phytase (Danisco Animal Nutrition, USA; 1000 FTU/kg; matrix values applied), and xylanase (Rovabio, Addisseo, France) was added at 100g/t. Birds were fed crumble forms of feed during the starter (d0-10) phase, while a pellet diet was fed during the grower (d10-24) and finisher (d24-35) phases. Feed ingredients were analyzed for nutrient composition using near-infrared spectroscopy (AminoNIR, Evonik Amino Prox, Essen, Germany), and the results were used to formulate a nutritionally balanced diet that meets the specifications recommended for Cobb 500 broilers (Cobb 500, 2022a). The formulated diet composition and nutrient content for each phase are presented in Table 2. Growth performance, mortality, liveability, flock uniformity, intestinal lesion scores, jejunal histomorphology, serum total antioxidant capacity, and malondialdehyde content from this experimental cohort have been reported previously (Khairunnesa et al., 2026). For the present study, a total of 120 birds were sampled. On the sampling days (d16 and d28), two birds were randomly selected from each of the 10 replicate pens per treatment, resulting in 20 birds per treatment and 60 birds at each sampling point and 120 sampled birds across both sampling days.

**Table 1:** Treatments applied in this study.

Treatments^1^	Additives	Inclusion level, (%)	NE^2^ Challenge
NC	-	N/A	Unchallenged
CC	-	N/A	Challenged
PHB	phytogenic blend	0.01	Challenged

NC^1^, non-challenged control; CC, challenged control; PHB, challenged control birds fed 0.01% phytogenic blend. NE^2^: necrotic enteritis.

**Table 2:** Diet composition (as-fed basis, %), and calculated nutrients.

			
Ingredients (%)	Starter (0-10 d)	Grower (10-24 d)	Finisher (24-35 d)
Corn	34.0	34.0	39.1
Soybean meal	34.0	28.5	22.8
Sorghum	15.2	20.2	20.3
Wheat	12.5	12.5	12.6
Dicalcium Phosphate	1.10	0.414	0.315
Limestone	1.09	1.10	1.04
Canola oil	0.300	0.867	1.280
Mineral Premix^1^	0.100	0.100	0.100
Vitamin Premix^2^	0.090	0.090	0.090
DL-methionine	0.320	0.310	0.306
L-lysine HCl 78.4	0.305	0.346	0.410
Salt	0.216	0.230	0.230
L-threonine	0.130	0.120	0.126
Choline Cl 70%	0.082	0.110	0.139
L-Arginine HCl	0.066	0.119	0.230
L-Valine	0.040	0.049	0.083
Na bicarbonate	0.020	0.020	0.024
Xylanase^3^	0.010	0.010	0.010
Phytase^4^	0.010	0.010	0.010
Sand^5^	0.419	0.960	0.871
Calculated nutrients^6^ (%, otherwise as indicated)			
Dry Matter	89.9	89.9	89.9
AME (kcal/kg)	2920	3010	3100
Crude protein	22.5	20.5	18.5
Crude fat	2.55	3.24	3.75
Crude fiber	3.00	2.90	2.85
Digestible Arg	1.36	1.25	1.18
Digestible Lys	1.26	1.16	1.08
Digestible Met	0.636	0.600	0.570
Digestible Meth+Cyst	0.940	0.881	0.826
Digestible Trp	0.286	0.257	0.226
Digestible Iso	0.855	0.765	0.668
Digestible Thr	0.867	0.780	0.705
Digestible Val	0.959	0.880	0.820
Calcium	0.960	0.800	0.740
Available Phosphorus	0.540	0.400	0.370
Sodium	0.170	0.170	0.170
Potassium	1.041	0.938	0.839
Chloride	0.243	0.265	0.284
Choline mg/kg	1710	1717	1715
Linoleic 18:2	0.913	1.02	1.15

AME=apparent metabolizable energy.

Trace mineral concentrate supplied per kilogram of diet^1^: Cu (sulfate), 16 mg; Fe (sulfate), 40 mg; I (iodide), 1.25 mg; Se (selenate), 0.3

mg; Mn (sulfate and oxide), 120 mg; Zn (sulfate and oxide), 100 mg; cereal-based carrier, 128 mg; mineral oil, 3.75 mg. Vitamin premix per kg diet^2^: vitamin A, 12 MIU; vitamin D, 5 MIU; vitamin E, 75 mg; vitamin K, 3 mg; nicotinic acid, 55 mg; pantothenic acid, 13 mg; folic acid, 2 mg; riboflavin, 8 mg; cyanocobalamin, 0.016 mg; biotin, 0.25 mg; pyridoxine, 5 mg; thiamine, 3 mg; antioxidant, 50 mg. Xylanase^3^: Rovabio (Addisseo, France) 100g/t. Phytase^4^: Axtra phytase gold (Danisco Animal Nutrition, USA), 1000 FTU/kg of diet. Sand was replaced with the required amount of PHB and added to the top^5^. Nutrient contents were measured using near-infrared spectroscopy^6^ (NIRS, Evonik Amino Prox, Germany).

### NE challenge

The present study followed a well-established NE challenge model (Rodgers et al., 2015; Wu et al., 2014). In brief, on d9, birds assigned to the challenge groups (CC and PHB) received an oral inoculation of 1 mL suspension containing 5000 *Eimeria acervulina*, 5000 *E. maxima*, and 2500 *E. brunetti* oocysts (Eimeria Pty Ltd., Ringwood, VIC, Australia) to induce coccidia infection as a predisposing factor of NE. Subsequently, on d14, the same group of birds received 1 mL of a *C. perfringens* (EHE-NE18) suspension (1 × 10^8^ CFU) via oral gavage (CSIRO Livestock, Geelong, VIC, Australia) to induce NE. Birds in the non-challenged control group received 1 mL of phosphate-buffered saline (**PBS**) on d9 and 1 mL of sterile thioglycolate broth on d14. All birds that died following the NE challenge were subjected to necropsy to confirm the cause of death. The occurrence and severity of the NE challenge in this experimental trial, including mortality, intestinal lesion scores, and performance outcomes, have been reported previously (Khairunnesa et al., 2026).

### Sample collection and preparation

On d16, two birds per pen were randomly selected, weighed, and humanely stunned using equipment from JF Poultry Equipment (Weltevreden Park, South Africa). Approximately 1 cm sections of the proximal jejunum were excised from each bird (2 birds per pen), placed individually into 2 mL Eppendorf tubes containing RNA later, and stored at −20°C until RNA extraction proceeded. Additionally, around 1 mL of cecal contents was collected from each bird, transferred into sterile 2 mL Eppendorf tubes, snap-frozen in liquid nitrogen, and stored at −20°C for subsequent DNA extraction. For microbiota profiling, caecal samples were collected separately from 2 birds/pen; microbiota data from the two birds were pooled during analysis to make a pen-level value (n = 10). Pooled cecal contents from the 2 sampled birds in each pen were also collected into sterile 2 mL tubes and stored at −20°C for SCFA analysis. The same sampling procedures were repeated on d28 for SCFA determination.

### Cecal contents for SCFA analysis

Cecal pooled samples were analyzed for SCFA using a method adapted from previously reported protocols (Gu et al., 2021; Richardson et al., 1989; Zhang et al., 2019). Briefly, approximately 450-600 mg of thawed cecal samples, previously stored at −20°C, were transferred into 2 mL tubes and kept on ice. To each tube, 1 mL of an internal standard solution (0.01 M 2-ethyl butyric acid and 0.05 M succinic acid-2,2,3,3-d_4_ in 0.2 M NaOH) was added. The mixture was vortexed for 5 minutes and then centrifuged at 12,000 g for 5 minutes. From the supernatant, 100 µL was transferred to a new 2 mL tube. Subsequently, 50 µL of 1 M HCl and 1.5 mL of diethyl ether were added, and the mixture was vortexed for another 5 minutes. The bottom aqueous layer was removed, and the diethyl ether was dried with anhydrous sodium sulfate. Then, 50 µL of the diethyl ether phase was transferred into a glass GC vial containing 400 µL of acetonitrile, to which 10 µL of dry pyridine and 25 µL of MTBSTFA solution (N‑tert-butyldimethylsilyl-N-methyl trifluoroacetamide, 1% t-BDMCS; N‑tert-butyldimethylchlorosilane) were added. The vial was promptly sealed and heated at 80°C for 30 minutes. The prepared samples were analyzed using gas chromatography-mass spectrometry (GC-MS; Agilent Technologies 7890A-5975C). Peaks were identified and quantified against known standards by utilizing single-ion chromatograms for the following ions: m/z 103 (formic acid), m/z 117 (acetic acid), m/z 131 (propionic acid), m/z 145 (iso- and n-butyric acids), m/z 158 (iso- and n-valeric acids), m/z 261 (lactic acid), m/z 289 (succinic acid), m/z 173 (2-ethyl butyric acid), and m/z 293 (succinic acid-2,2,3,3-d_4_). The concentrations of SCFA were expressed as µmol/g of digesta.

### RNA extraction and cDNA synthesis

Total RNA was extracted from jejunal tissue using the Bioline ISOLATE II RNA Mini Kit (Bioline Meridian Bioscience, Australia). Approximately 25-35 mg of tissue sample was placed into a 2 mL Eppendorf tube containing a 3 mm metal bead and homogenized in 350 µL of lysis buffer (Buffer RLY supplemented with 3.5 µL β-mercaptoethanol) using a Tissuelyser II (Qiagen, Hilden, Germany). The homogenate was then transferred onto a 2 mL ISOLATE II filter placed in a collection tube and centrifuged at 11,000 × g for 1 min. Subsequently, 350 µL of 70% ethanol was added, mixed thoroughly, and passed through an ISOLATE II mini-column by centrifugation at 11,000 × g for 30s . The column was transferred to a fresh collection tube, and 350 µL of Membrane Desalting Buffer (MEM) was added, followed by centrifugation at 11,000 × g for 1 min. Genomic DNA was removed by adding 95 µL of DNase I reaction mix (10 µL DNase I + 90 µL RDN buffer) to the membrane and incubating at room temperature for 15 min. The membrane was washed twice with wash buffers (200 µL RW1 and 600 µL RW2), each followed by centrifugation at 11,000 × g for 1 min. A final wash with 250 µL of RW2 was performed, followed by centrifugation at 11,000 × g for 2 min. RNA was eluted by adding 100 µL of nuclease-free water to the membrane and centrifuging at 11,000 × g for 1 min. Purified RNA was stored at −20°C. RNA concentration and purity were assessed using a NanoDrop ND-8000 spectrophotometer (Thermo Fisher Scientific, Waltham, MA), and RNA integrity was evaluated using an Agilent 2100 Bioanalyzer and RNA 6000 Nano Kit (Agilent Technologies, Inc., Waldron, Germany). Only samples with an A260/A230 ratio > 1.8, an A260/A280 ratio between 2.0 and 2.2, and an RNA Integrity Number (RIN) > 7.0 were used for downstream applications. For cDNA synthesis, the SensiFast cDNA synthesis kit (Bioline, Meridian Bioscience, Sydney, NSW, Australia) was used according to the manufacturer’s protocol. The RotorGene 6000 real-time PCR system (Corbett, Sydney, Australia) was used to perform reverse transcription at 42°C for 15 min, followed by a 3 min incubation at 95°C to convert RNA to cDNA. The resulting cDNA was diluted fivefold in nuclease-free water and stored at −20°C.

### Real-time quantitative polymerase chain reaction (RT-qPCR)

Quantitative real-time PCR was performed using the RotorGene 6000 system (Corbett, Sydney, Australia) and SensiFAST SYBR No-ROX Kit (Bioline, Sydney, Australia). Each 10 µL reaction contained 5 µL of 2× SYBR mix and 400 nM of each primer. Gene expression stability was assessed using the geNorm M module in qbase+ version 3.0 (Biogazelle, Zwijnbeke, Belgium), which assessed eight housekeeping genes: *glyceraldehyde 3-phosphate dehydrogenase* (*GAPDH*)*, 18S ribosomal RNA* (*RRN18S*)*, hydroxymethylbilane synthase* (*HMBS*)*, TATA box-binding protein* (***TBP***)*, tyrosine 3-monooxygenase/tryptophan 5-monooxygenase* (*YWHAZ*)*, Succinate Dehydrogenase Subunit A* (*SDHA*)*, Ribosomal protein L4* (***RPL4***)*,* and *β-actin* (*ACTB*). Based on M-values, *RPL4* (M-value = 0.261) and *TBP* (M-value = 0.261) were identified as the most stable reference genes for normalization. Amplification cycle (Cq) values were exported and analyzed using qbase+ software (Biogazelle, Zwijnbeke, Belgium), which employs an arithmetic mean approach to convert logarithmic Cq values into linear relative quantities by applying an exponential function for gene quantification (Hellemans et al., 2007; Vandesompele et al., 2002). Normalized relative quantities (NRQ) were calculated and analyzed across treatments. The specific primers used in this study are detailed in Table 3.

**Table 3:** Primer’s sequence used for quantitative real-time PCR.

Genes	Sequence (5′→3′)	Amplicon Size (bp)	Ta (°C)		Reference
Target genes					
*OCLN*	F-ACGGCAGCACCTACCTCAA R-GGGCGAAGAAGCAGATGAG	123	60	(Du et al., 2016)	
*JAM2*	F-AGACAGGAACAGGCAGTGCTAG R-ATCCAATCCCATTTGAGGCTAC	135	60	(Kumar et al., 2021)	
*CASP3*	F-TGGTGGAGGTGGAGGAGC R- GTTTCTCTGTATCTTGAAGCACCA	110	62	(Gharib-Naseri et al., 2020)	
*CLDN1*	F- CTTCATCATTGCAGGTCTGTCAG R- AAATCTGGTGTTAACGGGTGTG	103	60	(Zanu et al., 2020)	
*IgA*	F- GTCACCGTCACCTGGACACCA R- ACCGATGGTCTCCTTCACATC	192	64	(Lammers et al., 2010)	
*IgM*	F- GCATCAGCGTCACCGAAAGC R- TCCGCACTCCATCCTCTTGC	98	60	(Zhao et al., 2013)	
*MUC2*	F- CCCTGGAAGTAGAGGTGACTG R- TGACAAGCCATTGAAGGACA	143	60	(Fan et al., 2015)	
*IFN-γ*	F-AGCTGACGGTGGTGGACCTATTATT R- GGCTTTGCGCTGGATTC	259	60	(Li et al., 2018)	
*CLDN5*	F-GCAGGTCGCCAGAGATACAG R-CCACGAAGCCTCTCATAGCC	162	61	(Zanu et al., 2020)	
*b^0^****^,^****+AT*	F-CAGTAGTGAATTCTCTGAGTGTGAAGCT R-GCAATGATTGCCACAACTACCA	88	60	(Gilbert et al., 2007)	
*LAT1*	F-GATTGCAACGGGTGATGTGA R-CCCCACACCCACTTTTGTTT	70	60	(Gilbert et al., 2007)	
*APN*	F-AATACGCGCTCGAGAAAACC R-AGCGGGTACGCCGTGTT	70	60	(Gilbert et al., 2007)	
Reference genes					
*RPL4*	F: TTATGCCATCTGTTCTGCC R: GCGATTCCTCATCTTACCCT	235	60	(Yang et al., 2013)	
*TBP*	F-TAGCCCGATGATGCCGTAT R-GTTCCCTGTGTCGCTTGC	147	62	(Li et al., 2005)	

Gene^1^ name: *OCLN*: occluding; *JAM2*: junctional adhesion molecule 2; *CASP3*: Caspase-3; *IgA*: immunoglobulin A; *IgM*: immunoglobulin M; *MUC2*: Mucin 2; *bo’ +AT*: solute carrier family 7, member 9; *GLUT2*: glucose transporter-2; *IFN-γ*: Interferon-gamma; *LAT1*: L type amino acid transporter-1; *CLDN 5*: Claudin 5; *CLDN 1*: Claudin 1; *APN*: Aminopeptidase N; *TBP*: TATA-Box binding protein; *RPL4*: Ribosomal protein L4.

### Cecal DNA isolation

Cecal DNA was extracted from frozen samples collected on d16 using the QIAamp DNA Stool Kit (Qiagen, Hilden, Germany). Approximately 80 mg of cecal digesta was combined with 300 mg of BioSpec glass beads (0.1 mm) (Daintree Scientific, St Helens, Tasmania, Australia) in a 2 mL Eppendorf tube. To lyse bacterial cells, 500 μL of pre-warmed buffer ASL was added, and the sample was homogenized in a Tissuelyser II (Qiagen, Hilden, Germany) for 4 min at 30 cycles/s. The lysate was then incubated at 90°C for 10 min, vortexed for 15 s, and centrifuged at 20,000 × g for 1 min. The supernatant was transferred to a fresh 2 mL microcentrifuge tube, mixed with 300 μL of InhibitEx solution, and vortexed for 1 min. The mixture was then incubated at room temperature for 1 min to allow the inhibitors to bind to the InhibitEx matrix. After centrifugation at 20,000 × g for 3 min, 600 μL of the supernatant was transferred to a new tube containing 30 μL of Proteinase K. Subsequently, 600 μL of buffer AL was added, and the mixture was vortexed for 15 s, followed by incubation at 70°C for 10 min. Then, 500 μL of 96-100% ethanol was added, vortexed briefly, and centrifuged for 30 s. The lysate was passed through a spin column via centrifugation, and the column was washed sequentially with 500 μL of AW1 and AW2 buffers. For final elution, 100 μL of C6 buffer was added to the spin column, incubated at room temperature for 2 min, and centrifuged at 20,000 × g for 1 min. DNA concentration and purity were assessed using a NanoDrop 8000 spectrophotometer (Thermo Fisher Scientific, Waltham, MA), with samples showing an A260/A280 ratio > 1.8. Purified DNA was stored at −20°C for further analysis.

### Next-generation sequencing of cecal DNA samples

Amplicon sequencing targeting the V3-V4 region of the 16S rRNA gene was performed using dual-index primers pro341F (5′-CCTACGGGNBGCASCAG-3′) and pro805R (5′-GACTACNVGGGTATCTAATCC-3′), which included index sequences, heterogeneity spacers, and Illumina adapters (Fadrosh et al., 2014). Libraries were purified with AMPure XP beads (Beckman Coulter, USA) and sequenced on an Illumina MiSeq platform using a 2 × 300 bp paired-end protocol. Raw DNA sequences were demultiplexed using Cutadapt (Martin, 2011) and analyzed using Quantitative Insights into Microbial Ecology 2 (QIIME2) for microbiota analysis (Bolyen et al., 2019). Quality filtering, denoising, and chimaera removal were conducted using the Dada2 plugin (Callahan et al., 2016). Taxonomic classification was performed using the SILVA v138.1 reference database (Quast et al., 2012). A total of 1.8 million reads remained for the analysis, with an average of 30,000 reads per sample.

### Statistical analysis

All the data collected in this study were tested for normal distribution before statistical analysis. Except for the microbiota, the rest of the data were analyzed using JMP Pro, Version 18 (SAS Institute Inc., Cary, NC, USA), and Tukey's comparison test. Differences between means were considered statistically significant when the *P*-value was < 0.05 and were considered to show a tendency towards significance when 0.05 < *P* < 0.10. A pen was considered an experimental unit, with 10 replicates per treatment (n = 10). Downstream microbiome analyses and visualization were performed using R version 4.6.1 (R Core Team, 2026) in RStudio version 2026.08.2 + 200. Alpha diversity was assessed using the Shannon, Chao1, and ACE indices with the *vegan* package. Beta diversity was evaluated using Bray-Curtis and Jaccard dissimilarities with the *vegan* package. These differences were visualized using principal coordinate analysis (PCoA), and the permutational multivariate analysis of variance (PERMANOVA) further confirmed differences in microbial community composition between groups. Differentially abundant taxa were identified using LEfSe implemented with the *lefser* package. Relative-abundance profiles were visualized using *ggplot2* and *pheatmap*. Differences in bacterial relative abundance were assessed using the Kruskal-Wallis test followed by Benjamini-Hochberg (**BH**) correction using the *stats* package. Spearman correlation analysis was used to evaluate associations among bacterial taxa, and microbial association networks were visualized using *igraph* and *ggraph*.

## Results

### Cecal SCFA (d16 and d28)

On d16, dietary treatments significantly (*P* < 0.05) affected the concentrations of several SCFA in cecal digesta (Table 4). NE challenge significantly increased (*P* < 0.05) the concentrations of branched-chain fatty acids (**BCFAs**), iso-butyric, iso-valeric, and valeric acids compared to the NC treatment. Supplementation with PHB significantly (*P* < 0.05) reduced these branched-chain fatty acids compared to the CC treatment, showing no difference (*P* > 0.05) from the NC treatment. Lactic acid, succinic acid, and total SCFA concentrations did not differ significantly (*P* > 0.05) between the NC and CC groups. However, PHB supplementation significantly increased lactic acid and succinic acid (*P* < 0.05) compared to the CC treatment, with values comparable to those of the NC treatment (*P* > 0.05). Dietary PHB also significantly increased total SCFA compared to the CC treatment (*P* < 0.05). Additionally, the PHB group exhibited a tendency towards increased butyric acid concentrations compared to CC group (*P* = 0.097). By d28, no significant treatment effects were observed on cecal SCFA (*P* > 0.05) concentrations (Table 5).

**Table 4:** Effect of the phytogenic blend on d16 cecal SCFA of broilers challenged with necrotic enteritis.

Treatments^1^	Short-chain fatty acids concentration (µmol/g)									
	Formic	Acetic	Propanoic	Iso-butyric	Butyric	Iso-valeric	Valeric	Lactic	Succinic	Total SCFA
NC	1.71	33.3	1.70	0.186^b^	8.39	0.102^b^	0.410^b^	1.09^ab^	9.95^ab^	53.5^b^
CC	1.62	34.0	2.28	0.755^a^	9.71	0.590^a^	0.771^a^	0.869^b^	5.06^b^	55.6^b^
PHB	2.07	35.1	1.64	0.368^b^	12.5	0.217^b^	0.339^b^	6.47^a^	14.4^a^	73.0^a^
SEM^2^	0.256	3.17	0.227	0.0829	1.32	0.0797	0.0946	1.558	2.10	5.71
*P*-value	0.431	0.921	0.103	0.0002	0.097	0.0005	0.007	0.027	0.015	0.044

NC^1^, non-challenged control; CC, challenged control; PHB, NE-challenged birds fed 0.01% phytogenic blend; SCFA, short-chain fatty acids. ^ab^ values within a column with no common superscripts differ significantly (*P* < 0.05); SEM^2^, standard error mean.

**Table 5:** Effect of the phytogenic blend on d28 cecal SCFA of broilers challenged with necrotic enteritis.

Treatments^1^	Short-chain fatty acids concentration (µmol/g)									
	Formic	Acetic	Propanoic	Iso-butyric	Butyric	Iso-valeric	Valeric	Lactic	Succinic	Total SCFA
NC	0.959	48.1	3.51	0.442	6.49	0.205	0.620	1.36	5.93	67.6
CC	0.870	48.9	3.59	0.499	7.97	0.234	0.740	1.40	6.59	70.5
PHB	0.795	53.6	3.50	0.525	8.89	0.250	0.772	1.59	7.70	66.9
SEM^2^	0.0718	3.60	0.374	0.0449	0.892	0.0244	0.0578	0.195	1.362	6.76
*P*-value	0.277	0.533	0.980	0.420	0.178	0.427	0.166	0.655	0.655	0.922

NC^1^, non-challenged control; CC, challenged control; PHB, NE-challenged birds fed 0.01% phytogenic blend; SCFA, short-chain fatty acids. SEM^2^, standard error mean. No significant differences were observed at d28.

### Jejunal gene expression

The effects of dietary treatments on jejunal gene expression are summarized in Table 6. The NE challenge altered the expression of several genes related to immune response and barrier integrity. Compared with the NC group, the CC group showed significant upregulation of *IFN-γ* and *LAT1* (*P* < 0.05) and downregulation of *CLDN5* and *IgM* (*P* < 0.05). Supplementation with PHB did not show a significant difference (*P* > 0.05) from the CC group for these genes. Moreover, *LAT1\* showed an intermediate response in the PHB group and did not reach statistical significance compared to the CC group (*P* > 0.05). Although NE challenge had no significant (*P* > 0.05) effect on the expression of *APN* and *JAM2* between the CC and NC groups, *APN* expression was significantly higher (*P* < 0.05) in the PHB group compared to CC and NC. No significant differences (*P* > 0.05) were found among treatments for the expression of *CASP3, MUC2, OCLN, CLDN1*, and *bo+AT* genes.

**Table 6:** Effect of the phytogenic blend on d16 jejunal gene expression of broilers challenged with necrotic enteritis.

Treatment^1^	Gene name											
	*APN*	*CASP3*	*IFN-γ*	*JAM2*	*LAT1*	*MUC2*	*OCLN*	*CLDN1*	*CLDN5*	*IgA*	*IgM*	*bo+AT*
NC	0.913^b^	0.908	0.515^b^	1.17^a^	0.904^b^	1.03	0.949	1.09	1.26^a^	0.948	2.03^a^	1.23
CC	0.984^b^	1.20	1.94^a^	1.09^ab^	1.22^a^	1.07	1.02	0.953	0.955^b^	1.12	1.09^b^	0.935
PHB	1.35^a^	1.06	1.80^a^	0.869^b^	0.99^ab^	1.10	1.21	1.24	0.900^b^	1.14	0.727^b^	1.28
SEM^2^	0.1018	0.0896	0.2023	0.0731	0.0709	0.101	0.0879	0.1349	0.0598	0.0923	0.1967	0.1322
*P-value*	0.008	0.072	<.0001	0.014	0.009	0.885	0.104	0.339	<.0001	0.278	<.0001	0.155

NC^1^, non-challenged control; CC, challenged control; PHB, NE-challenged birds fed 0.01% phytogenic blend; ^ab^ values within a column with no common superscripts differ significantly (*P* < 0.05); SEM^2^, standard error mean.

### Cecal microbiota composition

#### Alpha diversity

The alpha diversity indices are presented in Fig. 1a–c. The Shannon diversity index (Fig. 1c) differed significantly among treatment groups (Welch's ANOVA, *P* < 0.001). Pairwise comparisons showed that the NC group had a higher Shannon index than both CC (*P* < 0.05) and PHB (*P* < 0.05), whereas no significant difference was observed between CC and PHB (*P* > 0.05). The Chao1 (Fig. 1a) and ACE (Fig. 1b) indices, which reflect species richness, did not differ significantly among groups (*P* > 0.05 for both).

**Fig. 1:**
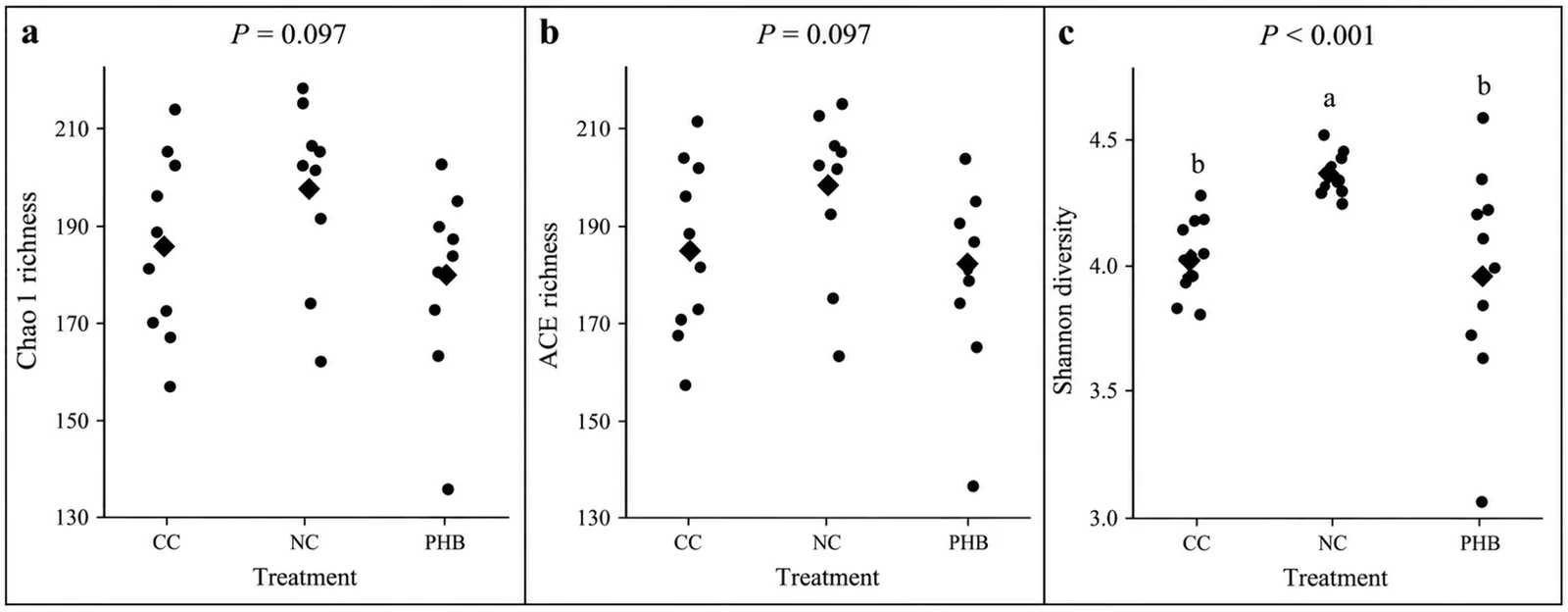
Effects of the phytogenic blend on the alpha diversity indices of the intestinal microbiota. (a) Chao1, (b) ACE, and (c) Shannon indices. NC = non-challenged control, CC = challenged control, PHB = NE-challenged birds fed 0.01% phytogenic blend. Statistical analysis was performed using one-way Welch’s ANOVA followed by Welch’s t-tests with Benjamini-Hochberg FDR correction (*P* < 0.05).

#### Beta diversity

Principal coordinate analysis (PCoA) based on Bray-Curtis and Jaccard distance matrices (Fig. 2a–c) showed significant differences in microbiota composition among treatment groups (permutational multivariate analysis of variance, **PERMANOVA**, F₂, ₂₇ = 2.651, R² = 0.164, *P* = 0.001; Jaccard: F₂, ₂₇ = 2.378, R² = 0.150, *P* = 0.001). For Bray-Curtis dissimilarity, pairwise comparisons revealed significant differences between NC and CC (*P* < 0.05), NC and PHB (*P* < 0.05), and CC and PHB (*P* < 0.01). For Jaccard dissimilarity, pairwise comparisons showed significant differences between CC and PHB (*P* < 0.01), whereas NC did not differ significantly from CC or PHB (*P* > 0.05for both).

**Fig. 2:**
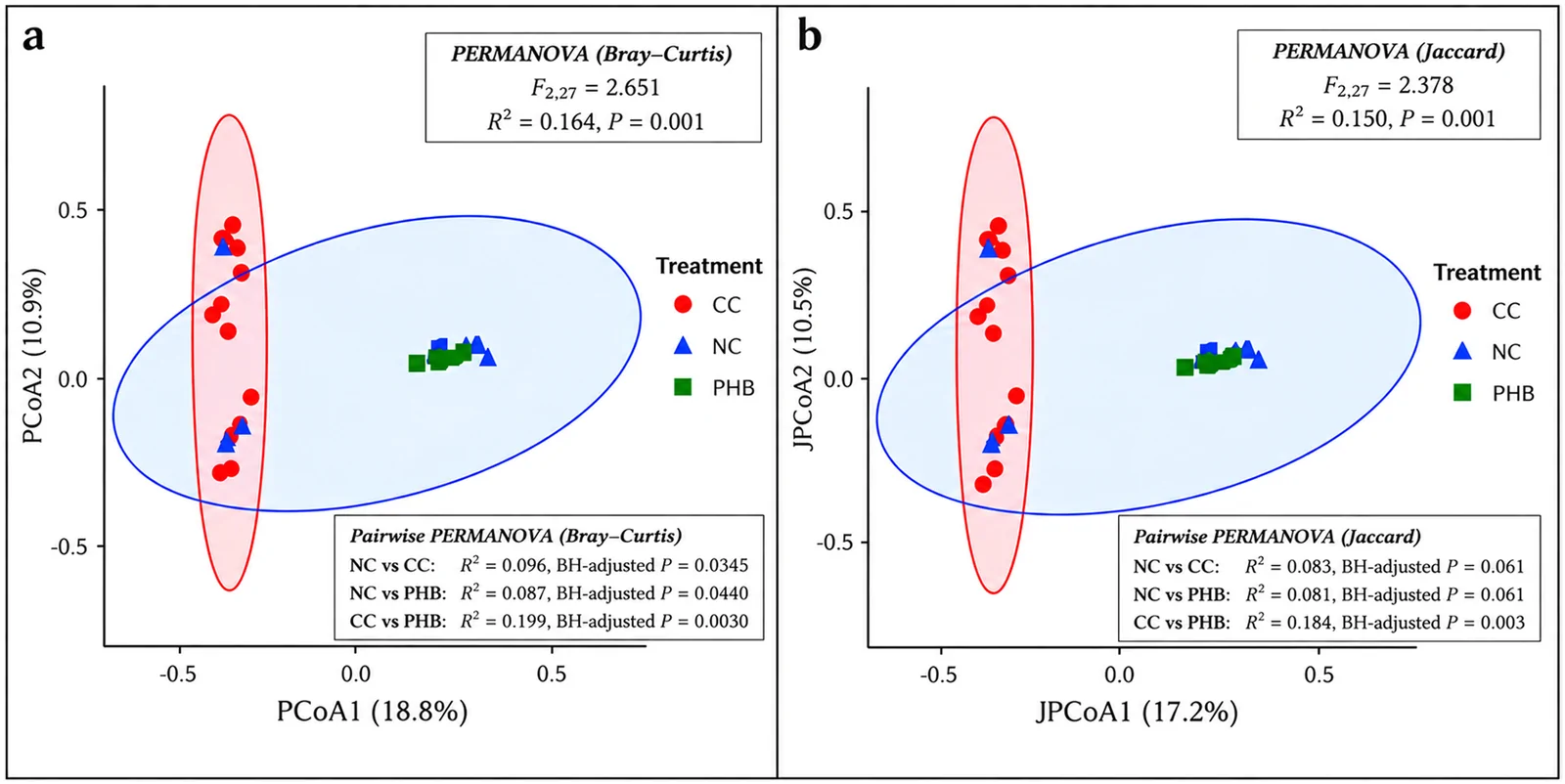
**Effect of the phytogenic blend on microbial community structure in broilers on d16**. Principal coordinates analysis (PCoA) based on (a) Bray-Curtis and (b) Jaccard dissimilarities. Each point represents an individual sample, and colored ellipses indicate treatment groups. NC = non-challenged control; CC = NE-challenged control; PHB = NE-challenged birds fed 0.01% phytogenic blend. Differences in microbial community composition were assessed using PERMANOVA with Benjamini-Hochberg (BH)-adjusted pairwise comparisons.

#### Differential bacterial abundance in treatment groups

The relative abundance of cecal microbiota at the phylum, family, and genus levels is illustrated in Fig. 3 (a-c). Microbiota analysis was conducted using 60 cecal DNA samples, comprising 10 replicate pens per treatment. At the phylum level, the dominant bacterial phyla across all treatment groups were Firmicutes, Proteobacteria, Bacteroidetes, and Actinobacteria (Fig. 3a). Firmicutes was the most dominant in all treatments, accounting for 98.4% in NC, 89.7.0% in CC, and 93.0% in PHB. In contrast, Proteobacteria were more abundant in challenged groups (0.74% in CC and 1.19% in PHB) compared to NC (0.26%). Similarly, *Bacteroidota* also became more abundant in challenged groups, rising from 1.2% in NC to 8.7% in CC and 5.1% in PHB. Actinobacteria abundance was low across all groups (< 1%).

**Fig. 3:**
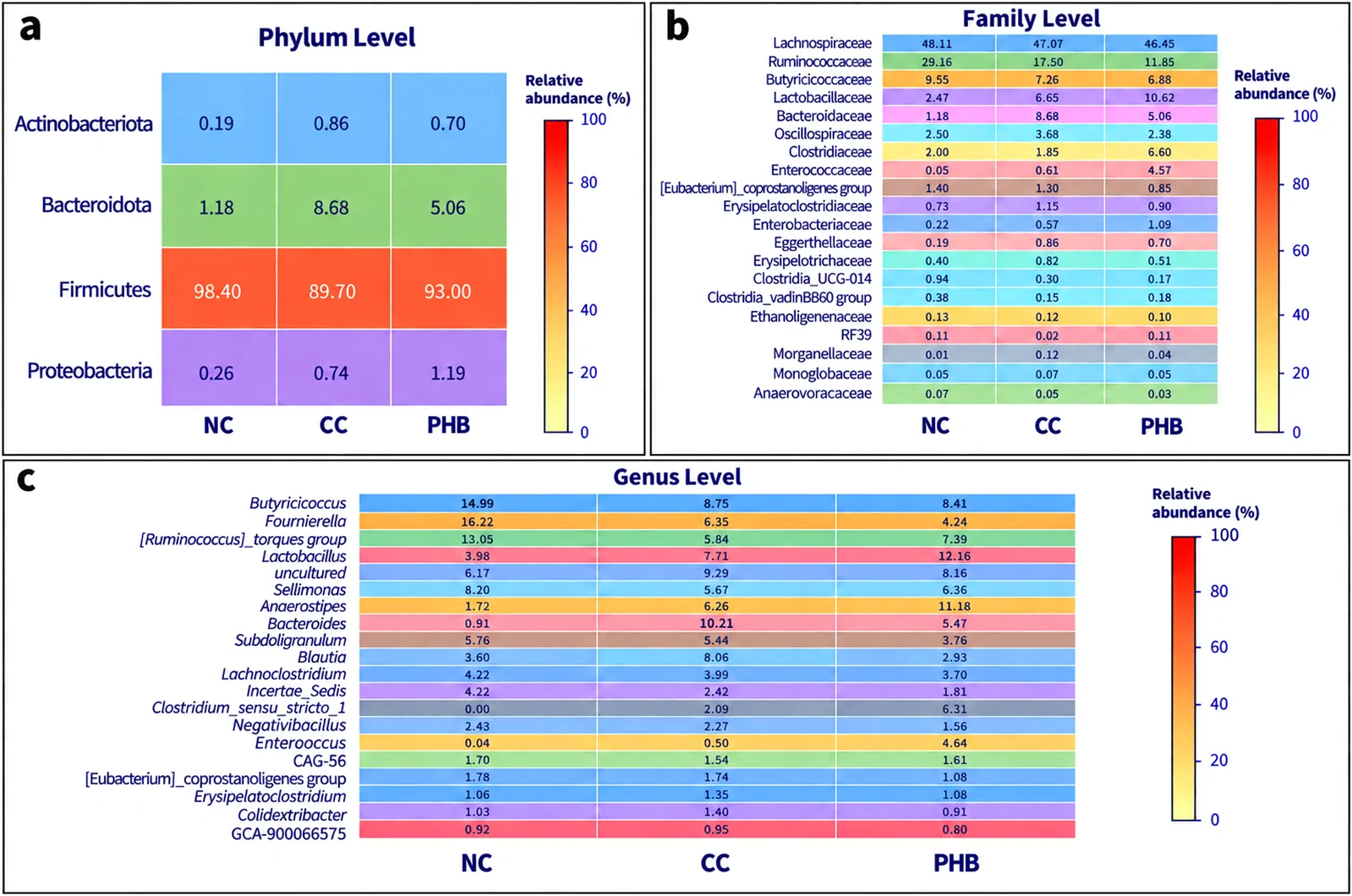
Relative abundance of cecal microbiota at different taxonomic levels in broilers on d16. Stacked bar plots show the relative abundance of dominant bacterial taxa. (a) phylum, (b) family, and (c) genus levels across the three treatment groups: NC = non-challenged control, CC = NE-challenged control, and PHB = NE-challenged birds fed 0.01% phytogenic blend. Each bar represents the mean relative abundance of taxa within a treatment group.

At the family level (Fig. 3b), Lachnospiraceae and Ruminococcaceae were the predominant families, with the highest relative abundance in NC 48.1.% and 29.2%, respectively. Both families showed lower relative abundance in the challenged groups, dropping to 47.1% and 17.5% in CC and 46.5% and 11.9% in the PHB group. Butyricicoccaceae also showed a lower abundance of 7.3% in CC and 6.9% in PHB compared to 9.6% in NC. In contrast, Lactobacillaceae appeared higher in challenged groups, increasing from 2.5% in NC to 6.7% in CC and 10.6% in PHB.

At the genus level (Fig. 3c), *Butyricicoccus,* and *Fournierella* were the predominant genera, with the highest abundance in NC at 15.0% and 16.2%, respectively. Their proportions were lower in the challenged birds, declining to 8.8% and 6.4% in CC and to 8.4% and 4.2% in PHB. *Ruminococcus_torques_*group showed a similar pattern, accounting for 13.1% in NC, 5.8% in CC, and 7.4% in PHB. In contrast, *Lactobacillus* increased from 4.0% in NC to 7.7% in CC and 12.2% in PHB. Similarly, *Anaerostipes* rose from 1.7% in NC to 6.3% in CC and 11.2% in PHB.

#### Linear discriminant analysis effect size (LEfSe)

LEfSe analysis (Fig. 4a–b) revealed differences in the relative abundance of cecal microbiota at the phylum and genus level among the treatment groups. At the phylum level, Firmicutes were enriched in NC, and Proteobacteria were predominant in both the CC and PHB groups. At the genus level, several taxa, including *Fournierella, Ruminococcus_torques_*group, *Butyricicoccus, Subdoligranulum, Sellimonas, Clostridia_vadin* BB60*_*group, *Oscillibacter, Clostridia*_*UGC_*014, and *Incertae sedis*, were enriched in the NC group. The CC group showed a higher relative abundance of *Blautia* and CHKCI002, whereas the PHB group showed greater abundance of *Lactobacillus, Anaerostipes, Clostridium sensu stricto* 1, *Enterococcus*, and *Escherichia Shigella*.

**Fig. 4:**
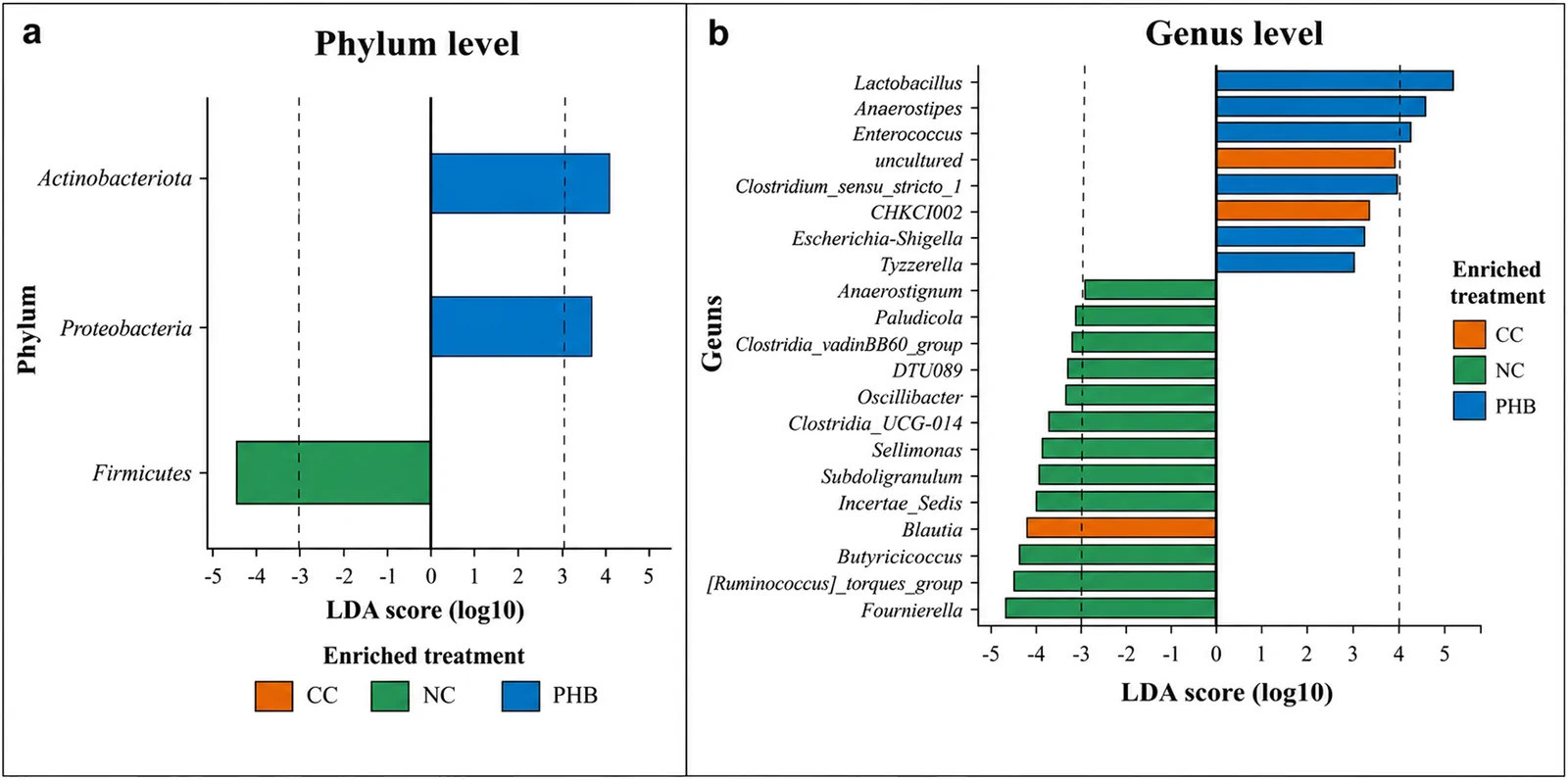
**LEfSe analysis of differential microbial taxa at the phylum (a) and genus (b) levels in broilers on d16**. Bars represent linear discriminant analysis (LDA) scores, with taxa enriched in each treatment shown in the corresponding color. Only taxa with LDA scores ≥3 are shown. NC = non-challenged control; CC = NE-challenged control; PHB = NE-challenged birds fed 0.01% phytogenic blend.

#### Univariate analysis

Univariate analysis (Kruskal-Wallis test followed by Benjamini-Hochberg; BH corrections) revealed that dietary treatments significantly influenced the relative abundance of 9 of the top 15 most abundant bacterial genera (BH-adjusted *P* < 0.05). Eight of these significant genera are illustrated in Fig. 5a–h. *Butyricicoccus, Fournierella, Ruminococcus_torques*_group, *Lactobacillus, Anaerostipes, Blautia, Clostridium sensu stricto* 1, *Incertae*_*Sedis*, and *Enterococcus* differed significantly among treatments (BH-adjusted *P* < 0.05). Compared with the NC group, the CC group had significantly lower abundances of *Butyricicoccus* and *Fournierella, Ruminococcus_torques*_group, and *Incertae_Sedis*, while having higher abundances of *Anaerostipes* and *Clostridium sensu stricto* 1 (BH-adjusted *P* < 0.05). *Lactobacillus* did not differ significantly between CC and NC. *Ruminococcus_torques*_group showed an intermediate response, but no significant difference either from NC or CC (BH-adjusted *P* > 0.05). Furthermore, the state of PHB remained unchanged for *Butyricicoccus, Fournierella, Clostridium Sensu stricto* 1, and *Incertae_sedis* in the CC group. The PHB group had higher *Lactobacillus* and *Anaerostipes* than NC, but no difference was observed between CC and NC (BH-adjusted *P* > 0.05). No significant treatment effects were detected for uncultured, *Bacteroides, Sellimonas, Subdoligranulum, Lachnoclostridium*, or *Negativibacillus* (BH-adjusted *P* > 0.05).

**Fig. 5:**
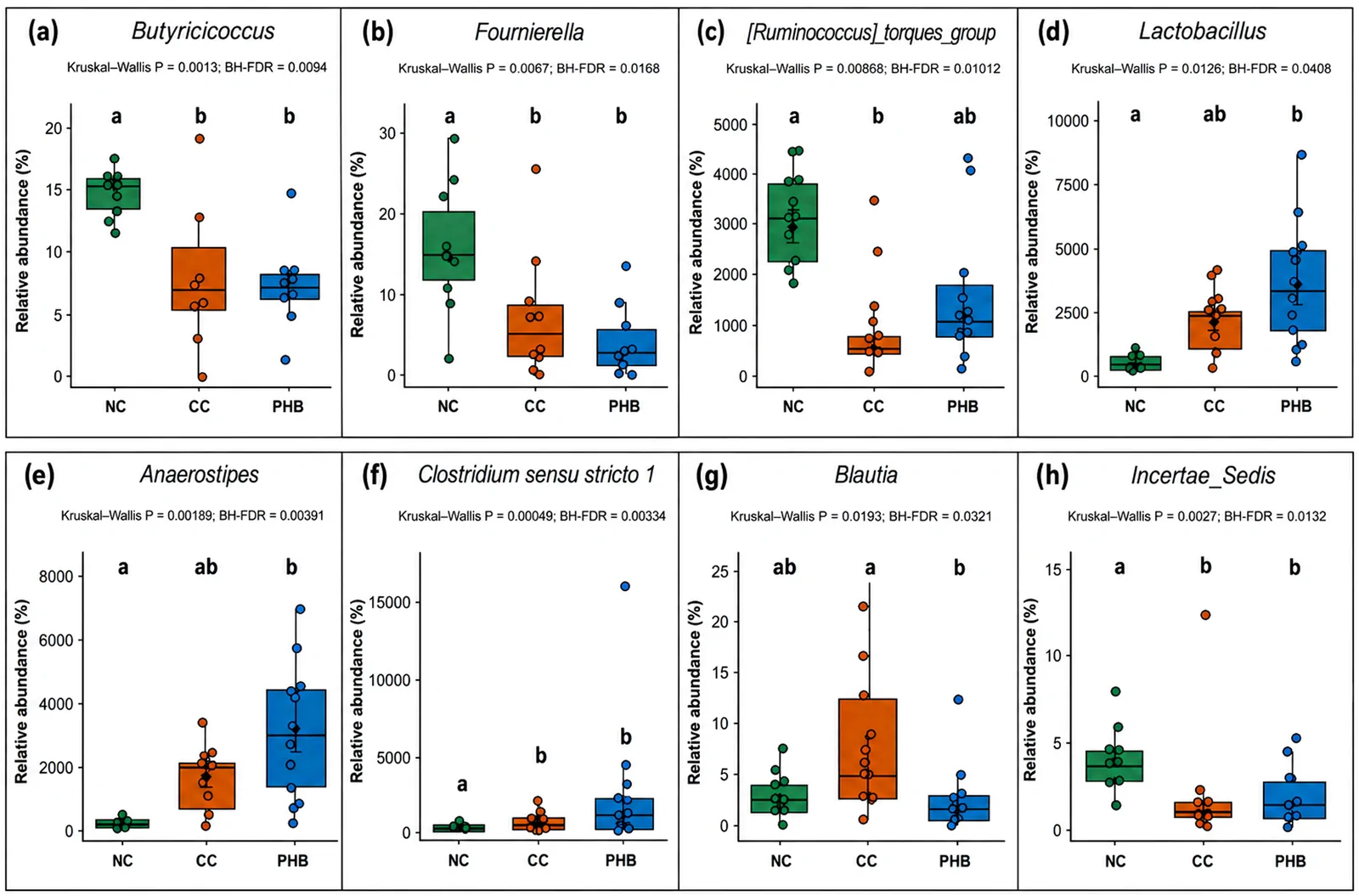
**Relative abundance of differentially abundant bacterial genera in broilers on d16**. (a) *Butyricicoccus*, (b) *Fournierella*, (c) [*Ruminococcus*]_*torques*_group, (d) *Lactobacillus*, (e) *Anaerostipes*, (f) *Clostridium sensu stricto* 1, (g) *Blautia*, and (h) *Incertae_Sedis*. Each point represents an individual pen (n = 10 per treatment). Different letters indicate significant differences among treatments based on BH-adjusted pairwise comparisons (P < 0.05). Kruskal-Wallis P-values and Benjamini-Hochberg (BH)-adjusted false discovery rates (FDR) are shown above each panel. NC = non-challenged control; CC = NE-challenged control; PHB = NE-challenged birds fed 0.01% phytogenic blend.

### Species-level correlation network analysis

Correlation network analysis at the species level (Fig. 6) revealed distinct microbial co-occurrence patterns among the detected microbial species. Spearman correlations were calculated across 30 pen-level observations, and associations with ∣ρ∣ ≥ 0.50 and *P* < 0.05 were visualized.

**Fig. 6:**
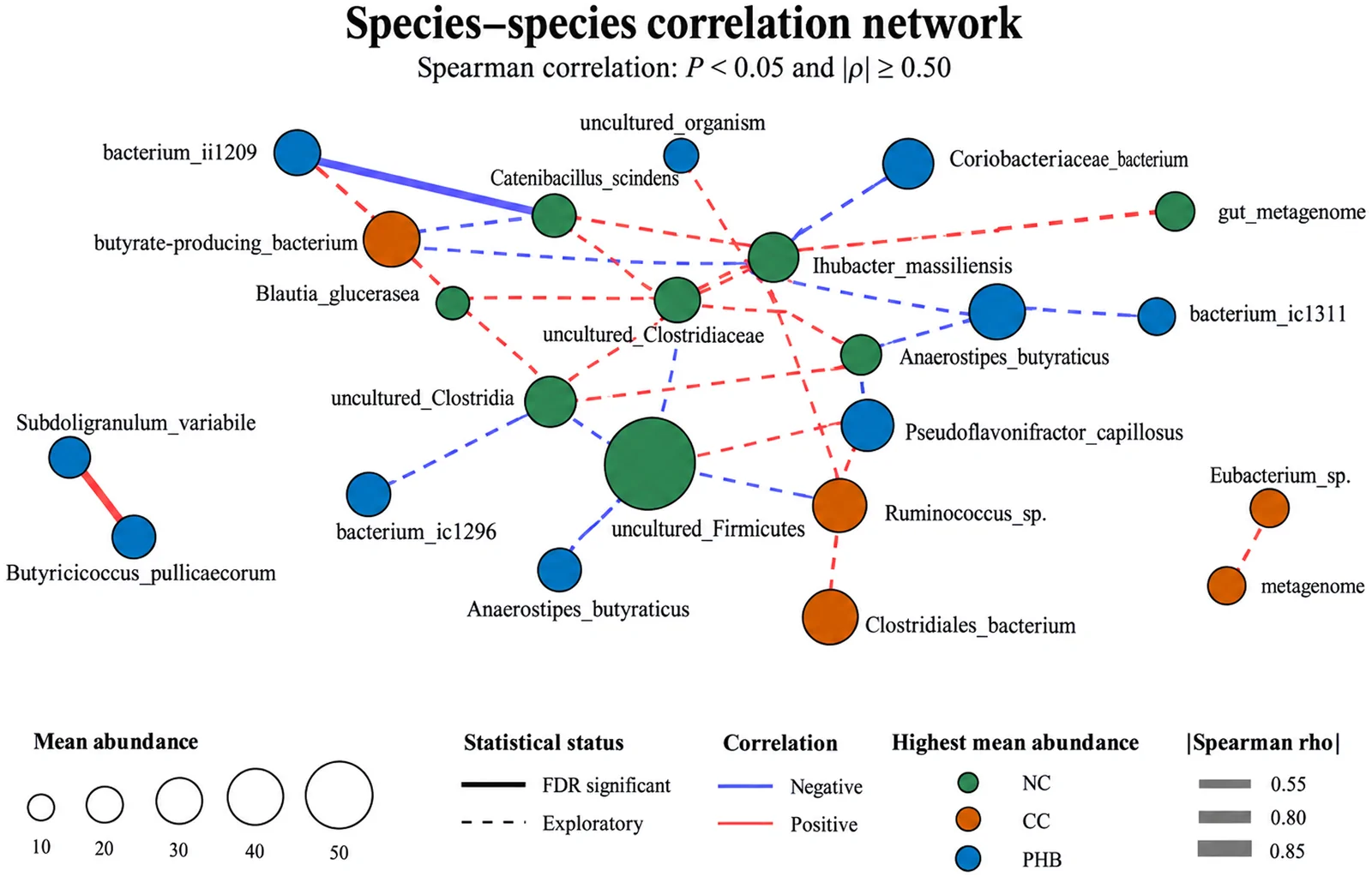
**Species-level Spearman correlation network of cecal microbiota on d16**. Associations were identified using Spearman correlation with P < 0.05 and |ρ| ≥ 0.50. Node size represents mean relative abundance, node color indicates the treatment with the highest mean abundance, and edge width represents the absolute Spearman correlation coefficient. Solid edges indicate associations significant after Benjamini-Hochberg false discovery rate (FDR) correction, whereas dashed edges indicate exploratory associations that were significant before FDR correction. NC= non-challenge, green nodes; CC= challenge control, orange nodes; PHB = NE challenged bird fed 0.01% phytogenic blend, blue nodes.

The red edges indicated positive and blue edges indicated negative associations, with solid lines for BH-FDR-significant links and dashed lines for exploratory links. The size of the node reflected relative mean abundance, while node color showed the treatment with the highest mean abundance for each species (NC, green; CC, orange; PHB, blue). The analysis consisted of 41 species, with 820 possible pairwise correlations. Several moderate-to-strong associations were identified; however, only two pairs remained significant after BH correction (BH-FDR < 0.05). Importantly, *Butyricicoccus pullicaecorum* had a significant positive correlation with *Subdoligranulum variabile* (ρ = 0.681, BH-FDR = 0.0278), which was detected in the PHB group, with a mean abundance of 13.0, compared to 0 in the NC and CC groups. The second significant correlation was a negative association between *bacterium_ii1209* (PHB group) and *Catenibacillus scindens* (NC; ρ =- 0.661, BH-FDR = 0.0285).

## Discussion

NE is a major threat to poultry health and production. Historically, NE was controlled with in-feed antibiotics, but concerns over antimicrobial resistance have limited their use. In this context, the present study investigated the effect of PHB, a phytogenic blend, on broilers challenged with NE*,* focusing on gene expression, SCFA production, and cecal microbiota modulation. This study extends our previous work (Khairunnesa et al., 2026), where the NE challenge was validated by reduced performance, increased intestinal lesions, oxidative damage, and impaired gut morphology. In our companion study (Khairunnesa et al., 2026), PHB supplementation improved d10-35 performance, reduced duodenal lesions, and improved villus height and crypt depth ratio compared to the CC group. Moreover, PHB shifted villus height, liveability, mortality, and excreta calprotectin and fibronectin biomarkers towards the NC group. In the present study, the NE challenge was further confirmed by reduced bacterial diversity (α and β diversity), increased abundance of harmful genera (*Clostridium sensu stricto* 1, *Escherichia Shigella*), and decreased beneficial genera abundance (*Ruminococcus*_*torques*_group, *Butyricicoccus*), coupled with upregulated inflammatory markers (*IFN-γ)* and downregulated gut barrier and immune-related genes (*CLDN5, IgM*), collectively indicating disrupted gut function. Dietary supplementation of PHB in the challenged birds showed positive effects on gut health, reflected by clear separation of CC and PHB groups in pairwise PERMANOVA, higher abundance of beneficial bacteria (*Tyzzerella, Anaerostipes*, and the *Ruminococcus_ torques_*group, *Lactobacillus*), upregulation of *APN* gene expression, reduced BCFAs, and elevated lactic, succinic, and total SCFA concentrations. Together, the improved performance and gut health in our companion study, along with these microbial, molecular, and metabolic responses, partially support the hypothesis that dietary PHB mitigates NE-induced intestinal damage by modulating microbiota, improving SCFA profiles, and regulating gene-associated nutrient absorption.

In this study, PHB supplementation led to distinct improvements in microbial, metabolic, and molecular parameters in NE-challenged broilers. Pairwise PERMANOVA based on Bray-Curtis and Jaccard dissimilarities showed clear separation of microbial community composition between the CC and PHB groups, indicating a shift in microbial community structure. A healthy gut microbiota is characterized by high microbial diversity and richness, which enhance nutrient absorption and utilization in chickens (Wei et al., 2022). Supplementation with PHB increased the abundance of several potentially beneficial taxa, including *Anaerostipes, Ruminococcus_ torque_*group, *Tyzzerella,* and *Lactobacillus*, which contribute to carbohydrate fermentation, SCFA production, and pathogen inhibition (Bhattacharjee et al., 2026; Cai et al., 2024; Oren et al., 2021). *Anaerostipes,* a member of the Lachnospiraceae family, and *Ruminococcus_ torques_*group (family Ruminococcaceae) are associated with the degradation and fermentation of complex carbohydrates. This fermentation process primarily produces acetate, butyrate, and propionate, which serve as key energy sources for the host and play a vital role in maintaining epithelial health (Biddle et al., 2013; Duncan et al., 2007; Liu et al., 2024; Louis et al., 2010). In line with these possible functions, PHB supplementation enhanced butyrogenic and lactic acid-producing bacteria. The increased abundance of *Tyzzerella* with PHB supplementation also may reflect a shift toward an SCFA-producing microbial community, as a previous study found increased abundance of this butyrate-producing bacterium with *Bacillus subtilis* supplementation or dietary intervention (Cai et al., 2024; Jia et al., 2024). Furthermore, species-level correlation network analysis provided further evidence of this microbial shift, identifying a higher abundance of butyrogenic bacteria, notably *Butyricicoccus* pullicaecorum, and its positive correlation with *Subdoligranulum variabile*, a bacterium associated with both lactic and butyric acid production (Duncan et al., 2007; Eeckhaut et al., 2008; Holmstrøm et al., 2004; Jia et al., 2022). The enrichment of *Lactobacillus* was also observed in the PHB group, aligning with previous findings on phytobiotic supplementation (Gilani et al., 2021; Islam et al., 2024; Sigolo et al., 2021). This response may be attributed to the synergistic antibacterial properties of PHB components (Yuan et al., 2023), since these likely suppressed pathogenic bacteria and facilitated the growth of beneficial microbes. *Lactobacillus* species produce bacteriocins and lactic acid, which help lower intestinal pH and inhibit acid-sensitive pathogens, including *C. perfringens* (Belenguer et al., 2007; Dec et al., 2014; Rani and Tiwari, 2023; Obianwuna et al., 2024). The 7.45-fold increase in lactic acid concentrations observed in the PHB group compared to CC further validates this mechanism. Along with the significant rise in lactic acid, PHB supplementation showed an increasing trend in butyrate, suggesting a more fermentative and health-promoting microbial profile. These results are consistent with previous studies, which have shown that phytogenic supplementation increased lactic and butyric acid (Dai et al., 2023; Liu et al., 2021). PHB supplementation also significantly reduced BCFAs (iso-butyric and iso-valeric), which are by-products of protein fermentation (Rios-Covian et al., 2020; Ríos-Covián et al., 2016; Smith and Macfarlane, 1997). The BCFAs and their nitrogenous toxic compounds can lead to higher intestinal pH, which can compromise gut health (Dąbek-Drobny et al., 2022; Rinttilä and Apajalahti, 2013; Russell et al., 2011). Thus, the reduction of BCFAs in the PHB group suggests a transition from proteolytic to saccharolytic fermentation, possibly leading to a lower intestinal pH that suppresses acid-sensitive pathogenic bacteria and favors a healthier gut environment (Apajalahti, 2005). Further evidence of increased saccharolytic activity was reflected in a 2.8-fold increase in succinate concentration in the PHB group compared to CC. Succinate acts as a metabolic precursor for propionate or butyrate, which contributes to better energy utilization and helps maintain mucosal integrity (Krautkramer et al., 2021; Louis and Flint, 2017; Reichardt et al., 2014). However, these changes were transient, as no significant differences in d28 cecal SCFA concentrations were observed across treatments. This may indicate recovery from the acute phase of NE as the birds aged. At the molecular level, NE challenge significantly downregulated *IgM* and *CLDN5*, while upregulating *IFN-γ* and *LAT1* genes. These transcriptional changes indicate impaired gut barrier function, increased inflammation, and altered nutrient transport (Dao et al., 2022; Gharib-Naseri et al., 2021, 2020; Park et al., 2008). However, PHB did not bring *IgM, CLDN5,* and *IFN-γ* genes back to normal levels. *APN*, a zinc-dependent brush border enzyme, catalyzes the final hydrolysis of peptides and facilitates amino acid absorption (Gal-Garber and Uni, 2000). The *APN* upregulation in the PHB group compared with both the NC and CC groups suggests that PHB may have enhanced intestinal peptide digestion and nutrient absorption, potentially contributing to the villus morphology observed in our companion study (Khairunnesa et al., 2026). In contrast, elevated *LAT1* expression in the CC birds may indicate a higher need for large neutral amino acids for epithelial repair and T-cell activation (Hayashi et al., 2013), and the intermediate *LAT1* expression in the PHB group may reflect reduced stress-induced amino acid transport activity. These results are consistent with previous NE challenge findings (Gharib-Naseri et al., 2021). The increasing tendency of *CASP3* in the CC group supports increased apoptosis, potentially mediated by *IFN-γ* (Cnop et al., 2005). In comparison, the intermediate *LAT1* expression in the PHB group indicates moderate metabolic activity and amino acid transport, which may reflect improved epithelial integrity and lower cell turnover. Collectively, these patterns of gene regulation suggest that PHB supplementation could help reduce NE-induced mucosal damage, support nutrient digestion and absorption, and possibly regulate intestinal inflammation. Together, these changes suggest PHB supplementation may alleviate NE-induced mucosal damage by enhancing nutrient digestion and shifting gut microbiota from a dysbiotic, proteolytic microbial profile under NE challenge to a more fermentative, SCFA-producing community.

Maintaining a healthy gut microbiota and balanced mucin dynamics is critical for intestinal health, particularly during NE challenge conditions. In the present study, the supplementation of PHB modulated key microbial taxa associated with mucin dynamics and gut stability. Interestingly, the relative abundance of the *R. torques* group, known for mucin-degrading activity in the human gut (Schaus et al., 2024), was also higher in the PHB group. While excessive mucin degradation is often considered detrimental, under NE challenge, it may have a beneficial role by preventing mucin overaccumulation, which otherwise serves as a nutrient source for *C. perfringens* (Yang et al., 2022). The presence of *R. torques* may therefore aid controlled mucin turnover, limiting substrate availability for *C. perfringens* and helping to maintain mucosal balance. The improvement of the Firmicutes-to-Bacteroidetes (F/B) ratio (increasing from 10.3 in CC to 18.4 in PHB; NC was 83.4) further indicates partial restoration of microbial stability, which may contribute to better nutrient utilization (Jumpertz et al., 2011; Keerqin et al., 2021; Turnbaugh et al., 2006). Overall, PHB supplementation appeared to modulate microbial composition, which may partly explain the improved gut health responses observed under NE challenge. *Butyricicoccus pullicaecorum* is an anaerobic, butyrate-producing bacterium first isolated from the caecum of broiler chickens and has since been recognized as a safe next-generation probiotic in humans, capable of enhancing gut SCFA production without disturbing the host microbiome (Boesmans et al., 2018; Eeckhaut et al., 2008). Species-level correlation network analysis in this study reveals that the butyrate producer *B. pullicaecorum* was exclusively present in the PHB group among the treatment groups and has a strong positive correlation with another butyrogenic bacterium, *Subdoligranulum variabile* (Holmstrøm et al., 2004). This finding aligns with earlier studies that have shown thymol-containing phytogenic blends increase butyric acid in broilers, supporting a microbial shift in the PHB group (Tiihonen et al., 2010; Yang et al., 2025). The PHB group in this study tended toward higher butyrate production and significantly higher lactate levels, indicating increased microbial cross-feeding activity. Even though butyrate-producing bacteria most likely use the lactate produced by primary fermenters as a substrate, the conversion of lactate to butyrate occurs gradually and might not be complete at the time of sampling d16, which could explain why the increase in butyrate was not significant. Furthermore, butyrate can be rapidly utilized at the epithelium, leading to low luminal concentrations at a single time point (Gasaly et al., 2021; Louis et al., 2022; Onrust et al., 2015). Importantly, *B. pullicaecorum* has previously been used as a probiotic strain in broilers, where its supplementation reduced harmful bacteria such as *Escherichia Shigella, Desulfovibrio*, and *Campylobacter*, improved FCR, and decreased the number of birds exhibiting NE lesions under challenge conditions (Eeckhaut et al., 2016). Beyond chickens, *B. pullicaecorum* enhanced butyrate-mediated anti-inflammatory and epithelial-protective responses by suppressing tumor progression and upregulating the SCFA transporter SLC5A8 and receptor GPR43 in a mouse model (Chang et al., 2020). Thus, these findings suggest that PHB supplementation promoted the proliferation of butyrogenic microbiota, contributing to a balanced and healthy gut environment, which in turn supported improved gut function and overall performance in broilers.

In conclusion, PHB supplementation alleviated NE-induced intestinal dysfunction by improving gut function, modulating beneficial microbiota, and regulating genes involved in nutrient digestion. These mechanistic findings are consistent with previous reports of improved performance, intestinal morphology, reduced intestinal lesion severity, and favorable shifts in mortality, liveability, flock uniformity, and calprotectin and fibronectin excreta biomarkers in the same experimental cohort (Khairunnesa et al., 2026), which together support the beneficial effects of PHB under NE challenge. These findings support the use of phytogenic feed additives as viable, non-antibiotic nutritional strategies for managing gut health in broiler production. Although this study provides mechanistic insight, further investigation into dose optimization and metabolomic pathways would strengthen the understanding of PHB functional mechanisms under NE challenge conditions. Microbiota profiling, gene expression, and SCFA were intentionally analyzed on d16 to capture the acute-phase response to the NE challenge, which was the primary focus of this study. Moreover, longitudinal assessment of the microbiota and intestinal gene expression in the later stage of production is recommended to assess the persistence of PHB-associated effects.

## Author contributions and roles

**Most Khairunnesa** contributed substantially to the study design and experimental planning, conducted the study and sample collection, performed laboratory analyses, analyzed and interpreted the data, and prepared the original manuscript draft. She critically reviewed and revised the manuscript for important intellectual content, reviewed and explicitly approved the final version of the manuscript.

**Alip Kumar** contributed substantially to the study design and experimental planning, supervised the research, contributed to the conduct of the study and sample collection, and participated in interpretation of the data. He critically reviewed and revised the manuscript for important intellectual content, reviewed and explicitly approved the final version of the manuscript.

**Shu-Biao Wu** contributed substantially to the conception and design of the study, supervised the research, and contributed to interpretation of the findings. He critically reviewed and revised the manuscript for important intellectual content, reviewed and explicitly approved the final version of the manuscript.

**Thi Thu Hao Van** performed the 16S rRNA sequencing and microbiota data processing and contributed substantially to the preparation and interpretation of the microbiota data. She critically reviewed and revised the manuscript, reviewed and explicitly approved the final version of the manuscript.

**Kowsig Palanisamy** contributed substantially to the study design and interpretation of the data and findings and provided funding for the research. He critically reviewed and revised the manuscript for important intellectual content, reviewed and explicitly approved the final version of the manuscript.

**Kosar Gharib-Naseri** contributed substantially to the conception and design of the study, provided overall scientific supervision throughout the study, managed the research funding, and contributed to data analysis and interpretation of the findings. She critically reviewed and revised the manuscript for important intellectual content, reviewed and explicitly approved the final version of the manuscript. All authors agree to be personally accountable for their own contributions and to ensure that questions related to the accuracy or integrity of any part of the work are appropriately investigated and resolved.

## Ethics declaration

This study was conducted in accordance with the ARRIVE (Animal Research: Reporting of In Vivo Experiments) guidelines. This study was approved by the University of New England.

## Disclosures

The authors declared that there are no conflicts of interest.
